# Inhibition of HIF-1*α* by the anticancer drug TAS106 enhances X-ray-induced apoptosis *in vitro* and *in vivo*

**DOI:** 10.1038/sj.bjc.6604720

**Published:** 2008-10-14

**Authors:** H Yasui, A Ogura, T Asanuma, A Matsuda, I Kashiwakura, M Kuwabara, O Inanami

**Affiliations:** 1 Laboratory of Radiation Biology, Department of Environmental Veterinary Sciences, Graduate School of Veterinary Medicine, Hokkaido University, N18 W9, Sapporo 060-0818, Japan; 2 Laboratory of Medicinal Chemistry, Graduate School of Pharmaceutical Sciences, Hokkaido University, N12 W6, Sapporo 060-0812, Japan; 3 Department of Radiological Technology, Hirosaki University School of Health Sciences, 66-1 Hon-cho, Hirosaki, 036-8564, Japan

**Keywords:** anticancer drug, apoptosis, HIF-1*α*, hypoxia, radiosensitisation, X-irradiation

## Abstract

In a previous study, we showed that a novel anticancer drug, 1-(3-C-ethynyl-*β*-D-*ribo*-pentofuranosyl)cytosine (TAS106, ECyd) increased the antitumour efficacy of X-irradiation. However, its effects on hypoxic cells in tumours remain unclarified. Here, we show that TAS106 enhances the induction of apoptosis in X-irradiated human gastric adenocarcinoma MKN45 and MKN28 cells under hypoxia *in vitro*. At the same time, the accumulation of HIF-1*α* observed under hypoxia was shown to be decreased to the level of normoxia in the presence of 0.1 *μ*M TAS106. To study the function of HIF-1*α* protein in apoptosis of hypoxic cells, we employed an HIF-1*α* reductive approach using its specific antisense oligodeoxynucleotide. The reduction of HIF-1*α* gene expression dramatically enhanced X-ray-induced apoptosis in hypoxic cells. In *in vivo* experiments in which MKN45 cells were transplanted into severe combined immunodeficient (SCID) mice, TAS106 (0.5 mg kg^−1^) suppressed HIF-1*α* expression and subsequently reduced the area of the hypoxic region in the tumour and enhanced the induction of apoptosis in the hypoxic region when combined with 2 Gy of X-irradiation. These results suggest the possibility that TAS106 acts as a potent radiosensitiser through the inhibition of HIF-1*α* expression and can be a useful agent against radiotherapy-resistant hypoxic cells in solid tumours.

The ribonucleoside anticancer drug 1-(3-C-ethynyl-*β*-D-ribo-pentofuranosyl)cytosine (TAS106, ECyd) shown in Figure 1A was first synthesised in 1995 and has been developed as a novel anticancer drug (Hattori *et al*, 1996). When TAS106 (ECyd) is ingested into tumour cells, it is rapidly phosphorylated to ECyd 5′-triphosphate, which has the potential to inhibit RNA synthesis because of its inhibition of RNA polymerase, leading to cell death (Shimamoto *et al*, 2002; Matsuda and Sasaki, 2004). Uridine/cytidine kinase (UCK) is a key enzyme for the first phosphorylation of TAS106 to ECyd 5′-monophosphate, generating cytotoxicity. The high activity of UCK in tumour cells relative to normal cells (Maehara *et al*, 1982; Koizumi *et al*, 2001; Matsuda and Sasaki, 2004) gives TAS106 an advantage in specificity for tumour therapy. In earlier studies, we have shown that both apoptosis and loss of clonogenic survival are induced through the abrogation of arrest at the G_2_/M phase in X-irradiated human (MKN45 and MKN28) and murine (Colon26) tumour cells, as well as Chinese hamster V79 normal cells, when X-irradiation is combined with TAS106 treatment *in vitro* (Inanami *et al*, 2004; Iizuka *et al*, 2005). The radiosensitising effects of TAS106 were also seen in colon26-transplanted tumours and MKN45 xenografts *in vivo* (Yasui *et al*, 2007). Interestingly, our previous study showed that repetitive treatments (three times at 2-day intervals) with the combination of X-irradiation and TAS106 induced tumour remission in half of the mice, whereas the treatment with X-irradiation alone or TAS106 alone showed only slight retardation of tumour growth without tumour remission. This result suggested the possibility that TAS106 radiosensitised hypoxic cells in addition to normoxic cells.

Hypoxia-inducible factor 1 (HIF-1) is a key mediator expressed in many cell types in response to oxygen deprivation. HIF-1*α*, an oxygen-sensitive HIF-1 subunit, plays an important role in protecting solid tumours against radio- and chemotherapy by promoting angiogenesis, increasing glucose metabolism and modulating apoptosis. In fact, Koukourakis *et al* and Vergis *et al* (Vergis *et al*, 2008) demonstrated that the overexpression of HIF-1*α* in squamous cell head-and-neck cancer and prostate cancer was associated with incomplete responses to surgery, chemotherapy, radiotherapy and chemoradiotherapy and with poor overall survival in clinical studies (Koukourakis *et al*, 2002). HIF-1*α* generally inhibits the induction of apoptosis by anticancer agents (Semenza, 2003; Kilic *et al*, 2007). It has been shown that HIF-1*α* exerts its antiapoptotic function by transcriptional activation of antiapoptotic proteins, that is, the Bcl-2 family and inhibitors of apoptosis (IAPs), such as survivin (Dong *et al*, 2003; Peng *et al*, 2006). In our recent studies, the inhibition of survivin expression by TAS106 was thought to mainly contribute to the radiosensitisation of apoptosis and the suppression of tumour growth *in vitro* and *in vivo* (Inanami *et al*, 2004; Yasui *et al*, 2007). Thus, it is possible to hypothesise that TAS106 controls HIF-1*α* expression and results in the suppression of antiapoptotic proteins, that is, its downstream transactivating factors.

To confirm the hypothesis described above, we examined the effects of TAS106 on X-irradiated tumour cells under hypoxia both *in vitro* and *in vivo* with special emphasis on the inhibition of HIF-1*α* expression.

## Materials and methods

### Materials

1-(3-C-ethynyl-*β*-D-*ribo*-pentofuranosyl)cytosine (TAS106) was synthesised as described elsewhere (Hattori *et al*, 1996). Ultrapure nitrogen gas (99.999%) was obtained from Air Water Technical Supply (Ishikari, Japan).

### Cell culture

Cells of human gastric adenocarcinoma cell lines MKN45 (TP53 wild-type) and MKN28 (TP53 mutation) were grown in RPMI 1640 medium (Gibco-BRL/Invitrogen, Carlsbad, CA, USA) supplemented with 10% fetal bovine serum (Filtron, Brooklyn, Australia) at 37°C in 5% CO_2_/95% air.

### Cell incubation, X-irradiation and drug treatment *in vitro*

Tumour cells attached to a 6-cm plastic dish were treated with TAS106 at the concentrations of 0.1 and 1.0 *μ*M for MKN45 and MKN28 cells, respectively, 1 h before hypoxic incubation. The hypoxic condition of tumour cells in the dish (oxygen concentration < 20 mmHg) was achieved by placing it in a gas-exchangeable chamber and continuously passing 95% N_2_/5% CO_2_ for 25 min on ice. The cells were then exposed to 20 Gy of X-rays while maintaining the gas flow. X-irradiation was performed with a Shimadzu PANTAK HF-350 X-ray generator (1.0 mm Al filter, 200 kVp, 20 mA, Shimadzu, Kyoto, Japan). After X-irradiation, the chamber was tightly closed and incubated at 37°C for the indicated periods. Cells under normoxia were also incubated for 25 min on ice and then exposed to X-rays. Hypoxia was also induced chemically by treating cells with 100 *μ*M CoCl_2_ for 12 h.

### RNAi to knockdown UCK2 gene in MKN45 cells

Annealed double-stranded siRNA (Stealth Select RNAi, Invitrogen, Carlsbad, CA, USA) for the *UCK2* gene as the follows: sense strand 5UAGCCGUUGAGACAGCCAUUGGUCU3 and antisense strand 5AGACCAAUGGCUGUCAACGGCU3. Transfection of MKN45 cells with the siRNA was performed using Lipofectamine™ 2000 according to the manufacturer’s instructions. As a negative control for the siRNA treatment, medium GC Stealth RNAi negative control duplex (MedGC, Invitrogen) was used.

### HIF-1*α* antisense oligonucleotide treatment of tumour cells

An HIF-1*α* antisense phosphorothioate oligodeoxynucleotide (AS-HIF-1*α*-ODN) and a scramble control (Srb-ODN) were synthesised by Sigma Genosys (Ishikari, Japan) according to published sequences (antisense sequence: 5′-GCCGGCGCCCTCCAT-3′; scramble sequence: 5′-ATGGAGGGCGCCGGC-3′; Zhang *et al*, 2004a). MKN45 cells were exposed to either Srb-ODN or AS-HIF-1*α*-ODN premixed with lipofectamine™ 2000. To assess the transfection efficiency, the 5′ ends of the oligonucleotides were conjugated to fluorescein-isothiocyanate (FITC). FITC-labeled oligonucleotides were transfected into MKN45 cells in the same conditions as above, and then the transfection efficiency was assessed by counting fluorescent cells.

### Fluorescence microscopic and flow cytometric observations of apoptotic cells

The detection of apoptotic cells was performed as described previously (Inanami *et al*, 2004). In brief, cells incubated for the indicated times after X-irradiation were collected by centrifugation. For fluorescence microscopy, cells fixed with 1% glutaraldehyde were stained with 40 *μ*g ml^−1^ propidium iodide (SIGMA Chemical Company, St Louis, MO, USA). Fluorescence microscopic observation was performed using an Olympus BX50 microscope (Olympus, Tokyo, Japan). The fraction of apoptotic cells was calculated as the percentage of apoptotic cells relative to the total cells observed microscopically. Three independent experiments were performed.

For flow cytometric analysis, tumour cells were collected at 48 h after X-irradiation and fixed with 70% ethanol. After being reacted with RNase A (Roche Diagnostics, Mannheim, Germany) for 30 min, the cells were stained with 50 *μ*g ml^−1^ propidium iodide. The DNA content in 10 000 cells was analysed using an EPICS ALTRA flow cytometer (BECKMAN COULTER, Fullerton, CA, USA).

### Clonogenic survival assay

The surviving fraction in MKN45 and MKN28 cells, which consisted of adherent cells and floating cells was assayed by using the plasma clot technique with platelet-poor human plasma according to the method described by Kashiwakura *et al* (Kashiwakura *et al*, 2002). In brief, the cells incubated with or without TAS106 for 24 h after X-irradiation were collected by trypsinization. Proper number (100–20 000) of tumour cells with 10% human platelet-poor AB plasma, the growth factor(s), penicillin (100 U ml^−1^), streptomycin (100 *μ*g ml^−1^), sodium pyruvate (1 mM), MEM vitamins (1%), MEM non-essential amino acids (1%), thioglycerol (10 *μ*M), L-asparagine (2 *μ*g ml^−1^), CaCl_2_ (74 *μ*g ml^−1^) and 0.2% bovine serum albumin in Iscove’s modified Dulbecco’s medium (Invitrogen) were plated in 24-well culture plates and incubated at 37°C in 5% CO_2_/95% air for 14 days (MKN45) and 10 days (MKN28). Under a microscope, colonies containing more than 50 cells were scored as surviving cells and the surviving fraction at each dose was calculated with respect to the plating efficiency of the non-irradiated control. The means of surviving fractions from three experiments were plotted to dose–response curves.

### SDS–PAGE and immunoblotting

Cells were collected at the indicated periods after X-irradiation and lysed in lysis buffer (25 mM Tris-HCl, pH 7.4; 300 mM NaCl; 10% glycerol; 3 mM EDTA; 1 mM MgCl_2_; 50 mM *β*-glycerophosphate; 25 mM NaF; 1% Triton X-100 and protease inhibitor cocktail). Immunoblot analysis was performed as previously described (Iizuka *et al*, 2005). The following antibodies were used: anti-HIF-1*α* (BD Transduction Laboratories, San Diego, CA, USA), anticaspase-3 (8G10; Cell Signaling Technology, Beverly, MA, USA), anti-UCK2 (ab37886; Abcam Inc., Cambridge, MA, USA), anti-cIAP2 (58C7; Cell Signaling Technology), antisurvivin (Alpha Diagnostic, San Antonio, TX, USA), anti-VEGF (Lab Vision Corp., Fremont, CA, USA) and antiactin (Santa Cruz Biotechnology). The bands were quantified by Image J software (National Institutes of Health, Bethesda, MD, USA).

### Semiquantitative reverse transcription–PCR (RT–PCR)

Total RNA was extracted and purified with an RNeasy Mini kit (Qiagen, Hilden, Germany) according to the manufacturer’s instructions. The specific primer sequences were as follows: for *HIF-1α*, 5′CCCCAGATTCAGGATCAGGATCAGACA3′ and 5′CCATCATGTTCCATTTTTCGC3′; for *UCK2*, 5′GCGAACCATGGCCGGGGACAGCGAG3′ and 5′ACAGTATGTACAGATGAGCAGTGCC3′; for *GAPDH*, 5′GAAGGTGAAGGTCGGAGTC3′ and 5′GAAGATGGTGATGGGATTTC3′. One microgram of RNA was reverse transcribed using the Reverse Transcription System (Promega Corporation, Madison, WI, USA) and amplification reactions were performed using GoTaq™ DNA Polymerase (Promega). The PCR protocol was as follows: initial denaturation at 95°C for 2 min, followed by 25 cycles at 95°C for 1 min, annealing at 53°C (HIF-1*α*) or 60°C (UCK2 and GAPDH) for 1 min and extension at 72°C for 1 min. The final extension was performed by an incubation step at 72°C for 5 min. The PCR products were subjected to electrophoresis in agarose gel and visualised with ethidium bromide.

### Animals and tumour models

C.B-17/Icr. SCID Jcl mice aged 6 weeks were purchased from Japan CLEA (Tokyo, Japan). MKN45 cells were subcutaneously injected into the footpad of the right hind leg (5 × 10^6^ cells per animal).

This study was conducted according to the established guidelines of the ‘Law for The Care and Welfare of Animals in Japan’ and approved by the Animal Experiment Committee of the Graduate School of Veterinary Medicine, Hokkaido University (approval no. 8092).

### Drug treatment and X-irradiation *in vivo*

When the tumour reached a size of 350 mm^3^, TAS106 administration and/or X-irradiation were performed only once. Animals were randomised into four groups: (1) no treatment, (2) X-irradiation (2 Gy) alone, (3) TAS106 administration alone and (4) TAS106 administration for 3 h followed by X-irradiation (2 Gy). TAS106 was i.p. injected into mice at the amount of 0.5 mg kg^−1^. Transplanted tumours were irradiated with a Shimadzu PANTAK HF-350 X-ray generator at a dose rate of 0.8 Gy min^−1^.

### Immunohistochemical analysis of hypoxic regions and HIF-1*α*

For the analysis of hypoxic regions, mice were treated with TAS106 administration and/or X-irradiation when the tumour reached a size of 350 mm^3^. The sizes of the treated tumours were monitored every day, and mice were sequentially killed when the tumour size reached 700 mm^3^. To visualise tumour hypoxia, mice were killed 90 min after i.p. injection of 60 mg kg^−1^ pimonidazole hydrochloride (Hypoxyprobe™-1 Kit; Chemicon International Inc., Temecula, CA, USA). Tumour tissues excised from mice were fixed in 4% buffered formaldehyde, embedded in paraffin and sectioned 5-*μ*m thick. The staining procedure for tumour hypoxia was according to the instructions of the Hypoxyprobe™-1 kit.

For HIF-1*α* immunostaining, the tumours were excised at 2 days after X-irradiation and/or TAS106 administration. Antigen retrieval was performed by pressure heating for 3 min in 0.01 M citrate buffer (pH 6.0). After quenching endogenous peroxidase, sections were incubated with an anti-HIF-1*α* antibody (NB100-654; Novus Biologicals, Littleton, CO, USA) diluted at 1 : 1000 overnight at 4°C. Then the slides were reacted with EnVision+ System-HRP labelled antirabbit polymer (K4002, Dako Cytomation, Kyoto, Japan). Immunoreactivity was visualised by incubation with DAB. Each stained slide was lightly counterstained with hematoxylin. The sections were analysed under an Olympus BX50 microscope.

### Immunofluorescent double staining for hypoxic cells and apoptotic cells

Tumour tissues excised from the mice at 2 days after X-irradiation and/or TAS106 administration were fixed with 4% paraformaldehyde, frozen using ultracold hexane and sectioned 8-*μ*m thick. After blocking non-specific binding sites, the slides were reacted with the appropriate dilution of hypoxyprobe™-1 MAb1 (1 : 500) and rabbit anticleaved caspase-3 (Asp175; Cell Signaling Technology; 1 : 1000) overnight at 4°C. The sections were incubated with Alexa Fluor 488 antimouse and Alexa Fluor 546 antirabbit secondary antibodies (Molecular Probes, Eugene, OR, USA). Then they were mounted on Prolong Gold antifade reagent with DAPI (Molecular Probes). Fluorescence microscopic observation was performed using an Olympus BX50 microscope with reflected light fluorescence.

### Scoring of stained sections

The hypoxic area was estimated by quantifying the ratio of the pimonidazole-positive area *vs* the total area of the slice after choosing a threshold using Scion Image software. Quantitative analysis for HIF-1*α* was performed by calculating the ratio of positively stained cells *vs* all tumour cells except for necrotic regions in five random fields at × 400 magnification. These quantifications were performed on at least three different xenografts in each treatment group.

### Statistical analysis

All results were expressed as the mean±s.e. The variance ratio was estimated by the *F*-test and differences in means of groups were determined by Student’s *t*-test or Welch’s *t*-test. The minimum level of significance was set at *P*<0.05.

## Results

### TAS106 enhanced radiation-induced apoptosis under hypoxia as well as normoxia in a UCK2-dependent manner in tumour cells

**Figure 1:**
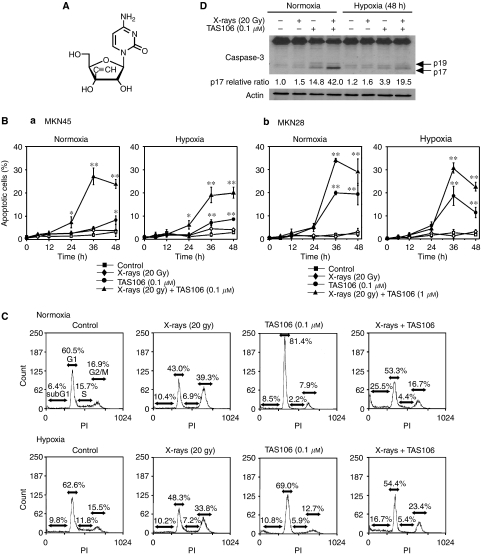
1-(3-C-ethynyl-*β*-D-*ribo*-pentofuranosyl)cytosine (TAS106, ECyd) enhances X-ray-induced apoptosis in MKN45 and MKN28 cells under normoxia and hypoxia. (**A**) The chemical structure of TAS106. (**B**) MKN45 (a) and MKN28 cells (b) treated with TAS106 (0.1 and 1.0 *μ*M for MKN45 and MKN28, respectively) and/or 20 Gy of X-irradiation were incubated for the indicated times under either normoxia or hypoxia. Apoptotic cells (%) were measured by fluorescence microscopy of cells stained with propidium iodide. Data are expressed as mean±s.e. for three experiments. ^*^P<0.05 *vs* control, ^**^P<0.01 *vs* control. (**C**) Cell cycle analysis of MKN45 cells treated with TAS106 and/or X-irradiation. After incubation under either normoxia or hypoxia for 48 h, cells were collected, fixed and stained with propidium iodide. The DNA content was measured by flow cytometry, and then the percentages of cell populations in the sub-G_1_ fractions of total cells were scored as mean±s.e. (**D**) Effects of X-irradiation and TAS106 treatment on the activation of caspase-3 in MKN45 cells. Tumour cells were collected at 48 h after each treatment. Arrows indicate the activated p19 and p17 fragments of casapse-3. Quantification of band of p17 fragments of casapse-3 was performed using Image J analysis normalised to actin.

Figure 1B (a) shows the effects of the combination of X-irradiation (20 Gy) and TAS106 (0.1 *μ*M) on apoptotic induction in MKN45 cells under normoxia and hypoxia. When MKN45 cells were treated with X-irradiation under normoxia or hypoxia, apoptotic induction was not observed until 48 h. Treatment of cells with TAS106 alone induced significant increases of apoptosis at 48 h under normoxia and at 36 and 48 h under hypoxia. However, the combined treatment with X-irradiation and TAS106 under normoxia significantly enhanced apoptosis in MKN45 cells from 24 h and 27.0±3.8% of cells were apoptotic at 36 h, similar to an erlier report (Inanami *et al*, 2004). A significant increase of apoptotic cells was also observed under hypoxia, although the apoptotic induction was less than that under normoxia (18.9±3.5% at 36 h).

Figure 1B (b) shows the effect of the combination of X-irradiation and TAS106 on apoptotic induction in MKN28 cells. Treatment of 0.1 *μ*M TAS106 could not enhance X-ray-induced apoptosis in MKN28 cells (data not shown), suggesting that MKN28 cells with TP53 mutation were more resistant to apoptotic induction by X-irradiation and TAS106 than MKN45 cells with wild-type TP53. Thus, 1.0 *μ*M was used as the dose of TAS106. TAS106 at this dose induced 20% apoptosis at 36 h under both normoxia and hypoxia. However, enhanced induction of apoptosis by combined treatment was observed compared to each treatment alone.

To study the relationship between the radiation-induced cell cycle checkpoint and the induction of apoptosis by TAS106, flow cytometric analysis was performed (Figure 1C). When MKN45 cells were exposed to 20 Gy of X-rays and incubated for 48 h under either normoxia or hypoxia, the flow cytometric profile showed a marked increase in the G_2_/M fraction but not in the sub-G_1_ fraction, suggesting the occurrence of radiation-induced G_2_/M arrest without apoptosis. In addition, the treatment of cells with TAS106 alone induced the accumulation of the G_1_ fraction, but not an increase in the sub-G_1_ fraction. However, the combination of X-irradiation and TAS106 resulted in a decrease in the G_2_/M fraction and an increase in the sub-G_1_ fraction (sub-G_1_ fraction; 10.4–25.5% under normoxia and 10.2–16.7% under hypoxia). A similar decrease in the G_2_/M fraction and increase in the sub-G_1_ fraction by TAS106 were also observed in irradiated MKN28 cells (data not shown). These results proved that TAS106 enhanced radiation-induced apoptosis under both normoxia and hypoxia regardless of the difference in the TP53 status.

As TAS106 was reported to enhance caspase-dependent apoptosis in X-irradiated tumour cells under normoxia (Inanami *et al*, 2004), we examined whether activation of caspase-3 was also induced by the combined treatment even under hypoxia. As shown in Figure 1D, immunoblot analysis revealed that X-irradiation combined with TAS106 treatment induced the expression of cleaved caspase-3 (p19 and p17, active forms of caspase-3), in MKN45 cells under normoxia and hypoxia. When tumour cells were treated with TAS106 alone or X-irradiation with TAS106 under hypoxia, the activation of caspase-3 was weaker than that under normoxia, suggesting that hypoxic cells were resistant to caspase-mediated apoptosis.

**Figure 2:**
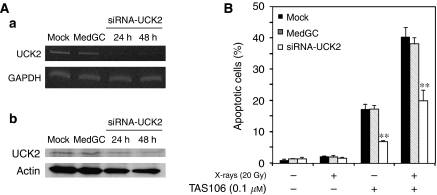
The enhancement of radiation-induced apoptosis by TAS106 is abrogated by UCK2 knockdown in MKN45 cells. (**A**) Semiconfluent MKN45 cells were incubated with double-stranded siRNA for the *UCK2* gene and lipofectamine™ 2000 in the absence of fetal bovine serum for 4 h and sequentially in the presence of fetal bovine serum for 20 h. As a negative control for the siRNA treatment, medium GC stealth RNAi negative control Duplex (MedGC) was transfected into tumour cells instead of siRNA. MKN45 cells were collected at 24 or 48 h after siRNA transfection, and then UCK2 expression at the mRNA level and protein level was examined by RT–PCR (a) and by immunoblotting (b), respectively. (**B**) After the siRNA transfection, MKN45 cells were treated with 0.1 *μ*M TAS106 and/or 20 Gy of X-irradiation. Apoptotic induction in tumour cells collected 36 h after each treatment was measured by fluorescence microscopy of cells stained with propidium iodide. Data are expressed as mean±s.e. for three experiments. ^**^P<0.01 *vs* mock control.

It has been reported that UCK2 is responsible for the phosphorylation of 3′-ethynyl nucleosides, including TAS106 (Murata *et al*, 2004), and this phosphorylation is essential for inhibition of RNA polymerase. Therefore, to determine the involvement of UCK2 in TAS106-induced apoptosis, we investigated whether TAS106 could enhance radiation-induced apoptosis in cells silenced *UCK2* gene by RNAi. UCK2 knockdown led to a reduction not only in UCK2 mRNA levels but also protein levels (Figure 2A). Silencing of UCK2 prevented the apoptotic induction by TAS106/X-irradiation treatment significantly compared to mock-transfected and MedGC-transfected cells (Figure 2B).

### Effects of TAS106 on cell survival examined by clonogenic ability

**Figure 3:**
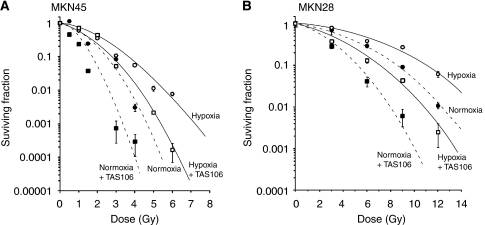
Dose–response curves of X-irradiated MKN45 (**A**) and MKN28 cells (**B**). Tumour cells were X-irradiated under normoxia (closed circles), normoxia+TAS106 (closed squares), hypoxia (open circles) and hypoxia + TAS106 (open squares; 0.1 *μ*M TAS106 for MKN45 and 1.0 *μ*M TAS106 for MKN28, respectively). Surviving fraction at each dose was calculated and corrected according to the plating efficiency of the non-irradiated control. Data are expressed as means±s.e. for three experiments.

We next examined a colony formation assay, which integrates multiple forms of cell death, such as apoptosis, necrosis and mitotic catastrophe, and hence gives overall cell killing that is correlated to tumour response *in vivo*. The survival curves in MKN45 cells exposed to X-rays under normoxia or hypoxia are shown in Figure 3A. In MKN45 cells without TAS106, the clonogenic ability in the cells exposed to X-rays under normoxia (10% survival; D_10_=2.5 Gy) was lower than that under hypoxia (D_10_=3.7 Gy). The treatment with 0.1 *μ*M TAS106 significantly enhanced the loss of clonogenic ability of MKN45 cells exposed to X-rays under normoxia and hypoxia (D_10_=1.75 Gy in normoxia+TAS106 and D_10_=2.75 Gy in hypoxia+TAS106). Furthermore, the clonogenic ability of MKN28 cells was also examined. As shown in Figure 3B, clonogenic abilities in MKN28 cells exposed to X-rays without TAS106 under normoxia (D_10_=8.4 Gy) and under hypoxia (D_10_=10.9 Gy) were significantly higher than those of MKN45 cells, respectively. This observation suggested that mutation of TP53 made MKN28 cells more resistant to X-irradiation. The treatment with 1.0 *μ*M TAS106 significantly enhanced the loss of clonogenic ability of MKN28 cells exposed to X-rays under normoxia and hypoxia (D_10_=5.1 Gy in normoxia+TAS106 and D_10_=6.9 Gy in hypoxia+TAS106), respectively.

### Effects of TAS106 on the expression of hypoxia-induced proteins related to apoptosis

**Figure 4:**
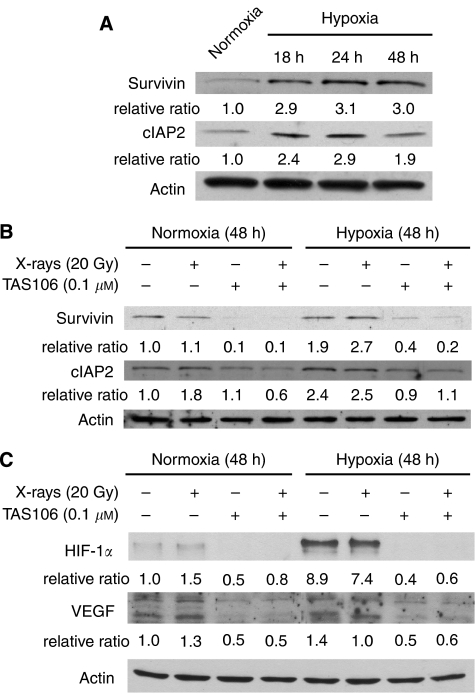
Immunoblots of the expression of survivin, cIAP2, HIF-1*α* and VEGF. (**A**) The time course of survivin and cIAP2 expression in MKN45 cells incubated under hypoxia for the indicated times. (**B**) Effects of TAS106 on the expression of survivin and cIAP2 under normoxia and hypoxia. MKN45 cells treated with 20 Gy of X-irradiation and/or 0.1 *μ*M TAS106 were incubated for 24 h. (**C**) Effects of TAS106 on the expression of HIF-1*α* and VEGF in MKN45 cells under normoxia and hypoxia. Tumour cells were incubated for 48 h after each treatment. Quantification of bands of survivin, cIAP2, HIF-1*α* and VEGF were performed using Image J analysis normalised to actin.

To gain insight into the modulation of apoptotic resistance in response to hypoxia in MKN45 cells, we next analysed the expression of IAP family proteins under hypoxia. As shown in Figure 4A, the expression of survivin and cIAP2 was increased under hypoxia and their upregulation reached the maximum level in MKN45 cells 24 h after hypoxic exposure. Furthermore, Figure 4B shows that X-irradiation did not affect survivin and cIAP2 expression, whereas 0.1 *μ*M TAS106 abrogated the upregulation of these antiapoptotic proteins under both normoxia and hypoxia.

### Effects of TAS106 on HIF-1*α* and VEGF expression in MKN45 cells under hypoxia

As hypoxia in solid tumours is a potent inducer of cell survival and angiogenesis through the upregulation of the HIF-1*α*-VEGF pathway, we investigated the effects of TAS106 on these hypoxia-related signalling molecules in MKN45 cells. As shown in Figure 4C, the hypoxic condition significantly increased the expression of HIF-1*α* up to 8.9-fold, whereas TAS106 treatment for 48 h completely inhibited its upregulation. The VEGF protein was constitutively expressed in MKN45 cells under both normoxia and hypoxia and was not changed by X-irradiation. However, its expression was markedly suppressed by treatment with TAS106 alone or X-irradiation with TAS106. These data clearly showed that TAS106 interfered with the upregulation of the HIF-1*α*-VEGF pathway under hypoxia.

### HIF-1*α* downregulation by TAS106 is largely dependent on transcriptional inhibition

**Figure 5:**
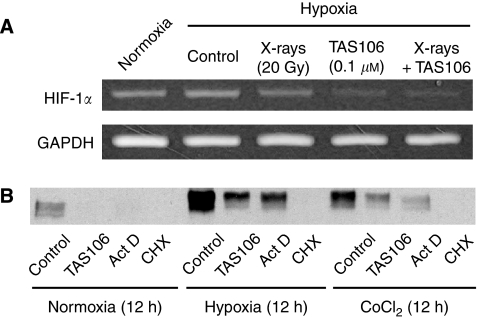
TAS106 suppressed the HIF-1*α* expression through its inhibitory effect on RNA synthesis in MKN45 cells. (**A**) MKN45 cells treated with 20 Gy of X-irradiation and/or 0.1 *μ*M TAS106 were incubated for 36 h under either normoxia or hypoxia, after which total mRNA was isolated and the expression of *HIF-1α* mRNA was analysed using RT–PCR. (**B**) After pretreatment with either 0.1 *μ*M TAS106, 25 ng ml^−1^ actinomycin D or 10 *μ*g ml^−1^ cycloheximide for 1 h, MKN45 cells were exposed to hypoxia or 100 *μ*M CoCl_2_ for 12 h in the presence of each drug. Cell lysates were prepared and subjected to immunoblot analysis to examine HIF-1*α* protein expression.

On the basis of its inhibitory effect on RNA polymerase, TAS106 is known to regulate the expression of its target proteins at the transcriptional level (Maehara *et al*, 1982; Hattori *et al*, 1996; Matsuda and Sasaki, 2004). In this study, we examined the effect of TAS106 on the mRNA expression of *HIF-1α* using semiquantitative RT–PCR. As shown in Figure 5A, hypoxic exposure for 36 h increased the mRNA expression of *HIF-1α* moderately; however, TAS106 treatment with or without X-irradiation obviously inhibited the mRNA expression of *HIF-1α*.

In addition, we compared the effects of TAS106, actinomycin D as an inhibitor of transcription and cycloheximide as a protein synthesis inhibitor on the expression of hypoxia- or CoCl_2_-induced HIF-1*α*. After MKN45 cells were exposed to hypoxia or CoCl_2_ in the presence of these agents for 12 h, cell lysates from tumour cells were used for immunoblot analysis. As shown in Figure 5B, 0.1 *μ*M TAS106 and 25 ng ml^−1^ actinomycin D had moderate inhibitory activities against the HIF-1*α* expression induced by both hypoxia and CoCl_2_, whereas 10 *μ*g ml^−1^ cycloheximide inhibited the expression of HIF-1*α* protein completely. Similar results were obtained from an experiment on HIF-1*α* overexpression using protease inhibitors, such as ALLN and MG132 (data not shown).

### Apoptotic induction by HIF-1*α* antisense oligonucleotide treatment in MKN45 cells under hypoxia

**Figure 6:**
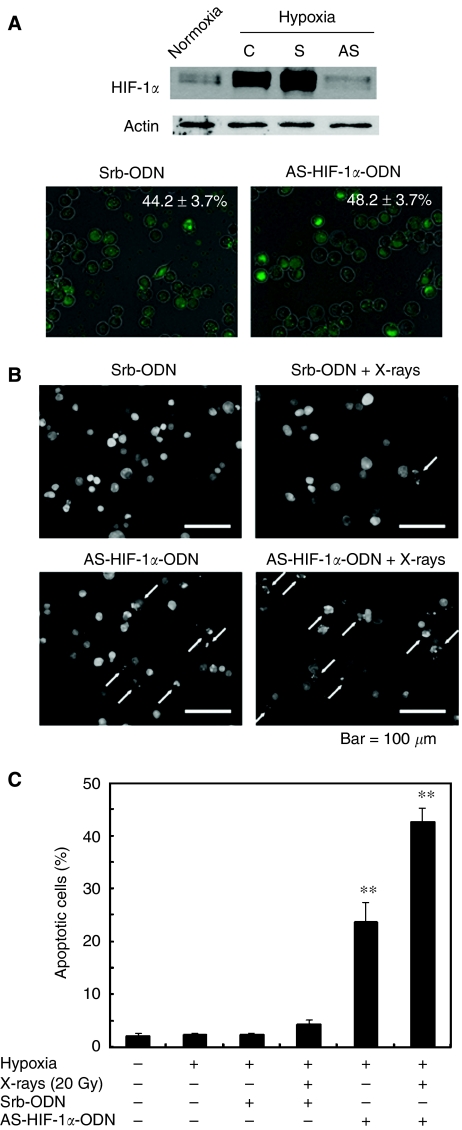
Effects of hypoxia and X-irradiation on apoptotic induction in MKN45 cells treated with an antisense HIF-1*α* oligodeoxynucleotide. (**A**) Hypoxia-induced HIF-1*α* expression was attenuated by an antisense HIF-1*α* oligodeoxynucleotide. Semiconfluent MKN45 cells were transfected with a scramble oligodeoxynucleotide (Srb-ODN; S) or antisense HIF-1*α* oligodeoxynucleotide (AS-HIF-1*α*-ODN; AS) for 24 h, followed by exposure to hypoxia for 3 h. To examine the transfection efficiency of Srb-ODN and AS-HIF-1*α*-ODN, FITC-labelled oligodeoxynucleotides were transfected as above, and the percentage of FITC-positive cells was measured by fluorescence microscopy. (**B**) Fluorescence microscopic observation of morphological changes in nuclei of cells treated with Srb-ODN alone, Srb-ODN and X-irradiation, AS-HIF-1*α*-ODN alone or AS-HIF-1*α*-ODN and X-irradiation. (**C**) Apoptotic induction in MKN45 cells under hypoxia treated with AS-HIF-1*α*-ODN and X-irradiation. After transfection with Srb-ODN or AS-HIF-1*α*-ODN followed by X-irradiation, MKN45 cells were incubated under hypoxia for 48 h. Apoptotic cells (%) were measured by fluorescence microscopy of cells stained with propidium iodide. Data are expressed as mean±s.e. for three experiments. ^**^P<0.01 *vs* control.

To confirm the role of HIF-1*α* protein in the apoptotic induction of MKN45 cells under hypoxia, we tried the HIF-1*α* reductive approach using its specific antisense ODN. First, to determine whether treatment with the antisense HIF-1*α* ODN (AS-HIF-1*α*-ODN) suppressed the expression of HIF-1*α*, MKN45 cells were transfected with AS-HIF-1*α*-ODN for 24 h, followed by incubation for 4 h under hypoxia. Figure 6A shows that hypoxia-induced HIF-1*α* expression was completely abolished by AS-HIF-1*α*-ODN. Transfection efficiency determined using FITC-labelled oligonucleotides was approximately 40–50% at 24 h after transfection for each ODN.

We next examined whether the reduction of HIF-1*α* affected apoptotic cell death of hypoxic cells. MKN45 cells were treated with either Srb-ODN or AS-HIF-1*α*-ODN and then cultured under hypoxia for an additional 48 h. Figure 6B shows fluorescence microscopic observation of the morphological changes in nuclei, including DNA fragmentation and chromatin condensation. Quantitative measurements of apoptotic induction revealed that the transfection of Srb-ODN did not increase the percentage of apoptotic MKN45 cells (2.4±0.1%) as compared with the mock-transfected control (2.2±0.4%; Figure 6C), whereas the transfection of AS-HIF-1*α*-ODN led to a significant increase in the induction of apoptotic cells (23.8±3.7%). Furthermore, combined treatment with AS-HIF-1*α*-ODN transfection and X-irradiation induced a marked increase in apoptosis (42.6±2.7%). These results proved that HIF-1*α* protected MKN45 cells against hypoxia-induced apoptosis and that the reduction of HIF-1*α* resulted in enhanced induction of X-ray-induced apoptosis in hypoxic cells.

### Reduction of the hypoxic area by combined treatment with X-irradiation and TAS106 in MKN45 xenograft

**Figure 7:**
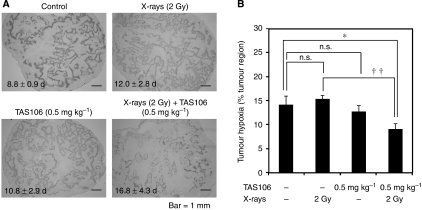
Effects of the combination of TAS106 (0.5 mg kg^−1^) and X-irradiation (2 Gy) on tumour hypoxia in MKN45 xenografts. When the tumour reached a size of 350 mm^3^, mice were treated with TAS106 administration and/or X-irradiation. The sizes of treated tumours were monitored every day, and then tumours were sequentially excised from mice when their sizes reached 700 mm^3^. (**A**) Typical photographs of MKN45 xenografts immunostained for pimonidazole. The number of days noted in each photograph shows the time required for tumour growth from 350 to 700 mm^3^. (**B**) The proportions of hypoxic areas in the whole tumours obtained by Scion Image software. Data are expressed as mean±s.e. for three different tumours. n.s.: *P*>0.05 *vs* control. ^*^P<0.05 *vs* control. ^††^*P*<0.01 X-irradiated group *vs* combination group.

In an erlier report (Yasui *et al*, 2007), we examined the *in vivo* antitumour efficacy of X-irradiation combined with TAS106 administration against two types of transplanted tumours (murine Colon26 and human MKN45 tumours). When 2 Gy of X-irradiation and low doses of TAS106 (0.1 and 0.5 mg kg^−1^ for colon26 and MKN45 tumours, respectively) were combined, significant inhibition of tumour growth was observed in both types of tumour compared with mice treated with X-irradiation and TAS106 alone. In this study, we first examined the growth delays of MKN45 xenografts treated with 2 Gy of X-irradiation and/or 0.5 mg kg^−1^ TAS106. The period it took for tumour volume to reach 700 mm^3^ from 350 mm^3^ was 8.8±0.9 days in the control group (Figure 7A). With 2 Gy of X-irradiation and 0.5 mg kg^−1^ TAS106, the time was delayed 3.2 and 2.0 days, respectively, whereas their combination delayed it 8.0 days. Next, to examine the effects of X-irradiation and/or TAS106 treatment on tumour hypoxia, the changes in the extents of the hypoxic areas in MKN45 xenografts were assessed by immunohistochemical analysis of the hypoxic marker pimonidazole. As shown in Figure 7A, obvious reduction of the hypoxic areas, that is, the pimonidazole-positive regions, were observed in tumours treated with the combination of X-irradiation and TAS106. The proportions of the hypoxic areas to the whole tumour regions were scored as 14.2±1.7% (control), 15.4±0.8% (X-irradiation alone) and 12.7±1.3% (TAS106 alone), indicating that a single treatment with TAS106 reduced the tumour hypoxic area moderately but not significantly (*P*>0.05; Figure 7B). However, in the case of the combination of X-irradiation and TAS106, the proportion of the hypoxic area significantly decreased to 9.1±1.1% compared with the control or X-irradiation alone (*P*<0.05). This result suggested that the combined treatment with X-irradiation and TAS106 significantly reduced the hypoxic area.

### Suppression of HIF-1*α* expression by TAS106 treatment *in vivo*

**Figure 8:**
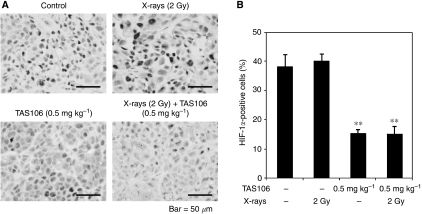
Suppression of HIF-1*α* expression by TAS106 treatment in MKN45 xenograft. Tumours were excised at 2 days after X-irradiation and/or TAS106 administration. (**A**) Typical microscopic fields of MKN45 xenografts immunostained for HIF-1*α*. (**B**) Quantitative data obtained by immunohistochemical analysis. Data are expressed as mean±s.e. for three different tumours. ^**^P<0.01 *vs* control.

To examine the effect of TAS106 on the expression of HIF-1*α* protein *in vivo*, we performed immunohistochemical analysis of HIF-1*α* (Figure 8A). Quantitative data showed that X-irradiation (2 Gy) alone had little effect on HIF-1*α* expression (40.0±2.4%) compared with the control (38.1±4.1%), but it was significantly suppressed to 15.2±1.3 and 15.1±2.6% in the tumours treated with TAS106 alone and the combination of X-irradiation and TAS106, respectively (Figure 8B). These results paralleled those shown in Figure 4C, suggesting that TAS106 also inhibited HIF-1*α* expression in MKN45 cells *in vivo*.

### Induction of apoptosis in hypoxic area by combined treatment with X-irradiation and TAS106 in MKN45 xenograft

**Figure 9:**
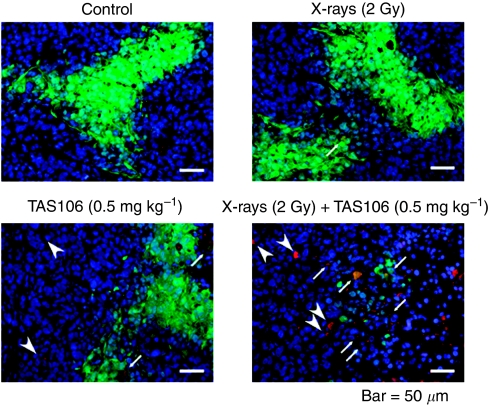
Induction of apoptosis in hypoxic cells of MKN45 xenografts by the combination of TAS106 (0.5 mg kg^−1^) and X-irradiation (2 Gy). Tumours were excised at 2 days after X-irradiation and/or TAS106 administration. Pimonidazole-positive cells (green) and cleaved caspase-3-positive cells (red) correspond to hypoxic cells and apoptotic cells, respectively. Tumour cells were also counterstained with DAPI (blue). Cleaved caspase-3-positive cells among normoxic cells (arrow head) and hypoxic cells (merged yellow, arrow) are observed in the MKN45 xenograft treated with X-irradiation and TAS106.

To investigate whether the combination of X-irradiation and TAS106 induced apoptosis in the hypoxic area, we conducted double-immunofluorescent staining for pimonidazole and cleaved casapse-3 using frozen tumour tissues excised 2 days after X-irradiation and/or TAS106 administration. Apoptotic cells were also observed in the DAPI-negative area corresponding to the necrotic region (Figure 9). In the control group and treatment group with either X-irradiation or TAS106 alone, few apoptotic cells were observed, among the pimonidazole-binding hypoxic cells. However, when tumours were treated with X-irradiation and TAS106, an obvious increase of apoptotic cells was detected in the hypoxic area.

## Discussion

We have previously shown that a wide-range inhibitor of RNA synthesis, TAS106, enhances X-ray-induced cell death regardless of the difference in TP53 status *in vitro* and *in vivo* (Inanami *et al*, 2004). The downregulation of X-ray-induced expression of survivin, a key molecule related to tumour survival and abrogation of the G_2_/M checkpoint because of the attenuation of CDC2-cyclin B1 kinase activity by TAS106 are responsible for this combined effect. Furthermore, we also demonstrated that low doses of TAS106 could potentiate the antitumour efficacy of X-irradiation with 2 Gy in both murine colon26 and human MKN45-transplanted tumours (Yasui *et al*, 2007). In particular, it was remarkable that repetitive treatments with the combination of X-irradiation and TAS106 induced tumour remission in half of the mice. On the basis of the biological activities of TAS106 and the tumour remission caused by the repetitive treatments, it is possible to assume that TAS106 also radiosensitises hypoxic cells in tumours.

Hypoxia is a common phenomenon in solid tumours because impaired vascular function results in an inadequate blood supply. As there is less X-ray-induced DNA damage in the absence of oxygen, hypoxic cells are resistant to killing by ionising radiation (Brown and Wilson, 2004). In addition, though severe or prolonged hypoxia initiates apoptosis, some cells adapt to hypoxia, survive and gain a more aggressive phenotype (Greijer and van der Wall, 2004). These tumour cells have reduced sensitivity to apoptosis and become more resistant to radiotherapy and chemotherapy. Therefore, it is of interest to investigate whether TAS106 enhances the radiation-induced apoptosis in hypoxic cells as well as in normoxic cells.

In the present *in vitro* studies, TAS106 enhanced radiation-induced apoptosis under normoxia and hypoxia in a TP53-independent manner, although the induction rate of apoptosis under hypoxia was smaller than that under normoxia (Figure 1B). As shown in Figure 1C, TAS106 abrogated the X-ray-induced G_2_/M checkpoint and increased the number of cells in the sub-G_1_ fraction under hypoxia in a fashion similar to that under normoxia. Furthermore, the combination of X-irradiation and TAS106 induced the activation of casapse-3 under both conditions, suggesting a TAS106-stimulated caspase-pathway leading to apoptosis (Figure 1D). This result supported our recent finding that the radiosensitisation of TAS106 was cancelled by the caspase inhibitor Z-VAD-fmk (Inanami *et al*, 2004). In addition, the present experiment using UCK2 knockdown by siRNA showed that the expression of *UCK2* gene in tumour cells was responsible for the enhancement of radiation-induced apoptosis (Figure 2B). This result supported the previous report that a 3′-ethynyl-nucleoside-resistant cell line established from human fibrosarcoma HT-1080 had a deletion of exon 4 of *UCK2* mRNA, resulting in a decrease of UCK2 expression (Murata *et al*, 2004). Interestingly, the induction of apoptosis by the combined treatment with X-irradiation and TAS106 was also observed in hypoxic cells in MKN45 xenografts, as shown in Figure 9. In addition to apoptosis induction, Iizuka *et al* demonstrated that TAS106 enhanced the loss of clonogenic ability by converting X-ray-induced proapoptotic cells into apoptotic cells in Chinese hamster V79 cells (Iizuka *et al*, 2005). When we examined the effect of TAS106 on clonogenic survival in MKN45 and MKN28 cells, TAS106 enhanced radiation-induced reproductive cell death not only normoxic condition but also hypoxic condition (Figure 3). These findings *in vitro* and *in vivo* strongly suggested that TAS106 could have potent activity to enhance the radiation-induced cell death of hypoxic cells.

For the application of TAS106 for clinical use, it is important to clarify the molecular mechanisms by which TAS106 could enhance the apoptotic induction of tumour cells with X-irradiation. As TAS106 is an inhibitor of RNA synthesis (Matsuda and Sasaki, 2004), we hypothesised that the expression of various apoptosis-related proteins would be non-specifically suppressed by TAS106. In this study, we tested the effect of TAS106 on the expression of anti- and proapoptotic proteins, such as IAPs and Bcl-2 family, in MKN45 cells under normoxia or hypoxia. As expected, the expressions of survivin and cIAP2 were effectively suppressed by the treatment of TAS106 under normoxia (Figure 4B). With respect to the role of survivin in resistance against radiation-induced apoptosis in tumour cells under normoxia, the overexpression of dominant-negative mutants of survivin (T34A and D53A) was demonstrated to enhance radiation-induced apoptosis in mouse fibroblast cell line NIH3T3, human cervical carcinoma cell line HeLa and human lung carcinoma cell line A549 (Ogura *et al*, 2008). The downregulation of both survivin mRNA and protein expression by TAS106 led to the enhancement of X-ray-induced apoptosis and overexpression of wild-type survivin in MKN45 cells inhibited this apoptotic induction (Inanami *et al*, 2004). These results indicated that survivin played an important role in the radioresistance of tumour cells under normoxia. Interestingly, it has been shown that the resistance of hypoxic cells to apoptosis is modulated through the upregulation of IAPs, including survivin and cIAP2, induced by hypoxia (Semenza, 2003). In this study, the expression of survivin and cIAP2 was increased in MKN45 cells under hypoxia for 24 h (Figure 4A), suggesting that this upregulation provided MKN45 cells with the resistance to apoptosis observed in Figure 1B and 1C. As shown in Figure 4B, TAS106 reduced the expression of survivin and cIAP2 upregulated by hypoxic exposure, indicating that the downregulation of these antiapoptotic proteins by TAS106 also contributed to the enhancement of radiation-induced apoptosis of hypoxic cells as well as normoxia. Furthermore, we examined whether TAS106 modulated the Bcl-2 family, which is the major regulator of caspase activation. The Upregulation of Bax and downregulation of Bcl-2 favour the proapoptotic response over the antiapoptotic one in tumour cells, leading to the release of cytochrome c and promotion of cell death (Cory and Adams, 2002). In this study, TAS106 treatment reduced the expression of both antiapoptotic Bcl-2 and proapoptotic Bax proteins under both normoxia and hypoxia (data not shown). Thus, TAS106 could not increase the Bax/Bcl-2 ratio in MKN45 cells, suggesting that it might not make the proapoptotic response dominant over the antiapoptotic one.

It is noteworthy that immunoblot analysis and immunohistochemical analysis revealed that TAS106 treatment suppressed the expression of HIF-1*α* proteins *in vitro* and *in vivo* (Figures 4C and 8). Concerning the mechanism of the HIF-1*α* suppression by TAS106, semiquantitative RT–PCR showed that TAS106 inhibited the mRNA expression of HIF-1*α* in MKN45 cells under hypoxia (Figure 5A). In addition, the pattern of inhibition of HIF-1*α* protein by TAS106 was similar to that of actinomycin D, a transcription inhibitor. These results suggested that TAS106 downregulated HIF-1*α* expression transcriptionally as well as actinomycin D. In addition, treatment with cycloheximide, a protein synthesis inhibitor, also inhibits hypoxia-induced HIF-1*α* expression. The adaptational response of tumour cells against hypoxia is thought to be mainly mediated by HIF-1*α*, which transactivates a number of target genes leading to metabolic adaptation, angiogenesis, invasion/metastasis and apoptosis resistance (Semenza, 2003). Recent studies reported that interfering treatments targeting HIF-1*α,* such as antisense or siRNA, induced apoptosis of human tongue squamous carcinoma cells *in vitro* (Zhang *et al*, 2004a) and inhibited tumour growth in human cervical and colon adenocarcinoma cells *in vivo* (Zhang *et al*, 2004b). In this experiment, we demonstrated that the reduction of the constitutive level of HIF-1*α* by an antisense HIF-1*α* oligonucleotide markedly increased the number of apoptotic cells (Figure 6C). Zhang *et al* (2004a) demonstrated that the abrogation of both basal and hypoxia-induced HIF-1*α* protein levels induced apoptosis under not only hypoxia but also normoxia in squamous carcinoma cells with constitutive HIF-1*α* expression. Combined with their result, our findings suggest that maintaining a constitutive level of HIF-1*α* is essential to cell survival. Interestingly, we demonstrated that X-irradiation enhanced HIF-1*α* reduction-induced apoptosis (Figure 6C). These data paralleled the synergistic effect of TAS106 on X-ray-induced apoptosis as shown in Figure 1B, suggesting that TAS106 radiosensitised cells to apoptotic induction through the suppression of HIF-1*α* expression. Recent reports have shown that survivin gene expression might be regulated by hypoxia and subsequent activation of HIF-1*α* (Dong *et al*, 2003; Greijer and van der Wall, 2004; Peng *et al*, 2006; Kilic *et al*, 2007). In this experiment, survivin expression increased under hypoxia for 24 h, suggesting a linkage to hypoxia-induced HIF-1*α* expression. Therefore, it is possible that the apoptotic resistance of hypoxic cells was abrogated through the inhibition of HIF-1*α*-induced antiapoptotic molecules by TAS106. Further information is necessary to clarify the mechanism by which TAS106 overcomes the resistance of hypoxic cells to X-ray-induced apoptosis.

In conclusion, we demonstrated that TAS106 could radiosensitise apoptotic induction in not only normoxic but also hypoxic cells *in vitro* and *in vivo*, which might be mainly related to the inhibition of HIF-1*α* expression. Importantly, TAS106 significantly reduced the hypoxic regions of X-irradiated MKN45 xenografts, probably because of this apoptotic induction. To overcome the radioresistance of apoptosis in hypoxic cells of solid tumours, the use of radiation sensitisers against not only normoxic cells but also hypoxic cells is necessary in combination with radiotherapy. On the basis of our results, the novel anticancer drug TAS106, which was effective for hypoxic cells through HIF-1*α* inhibition is a candidate sensitiser for radiotherapy.

## References

[bib1] Brown JM, Wilson WR (2004) Exploiting tumour hypoxia in cancer treatment. Nat Rev Cancer 4: 437–447 15170446 10.1038/nrc1367

[bib2] Cory S, Adams JM (2002) The Bcl2 family: regulators of the cellular life-or-death switch. Nat Rev Cancer 2: 647–656 12209154 10.1038/nrc883

[bib3] Dong Z, Wang JZ, Yu F, Venkatachalam MA (2003) Apoptosis-resistance of hypoxic cells: multiple factors involved and a role for IAP-2. Am J Pathol 163: 663–671 12875985 10.1016/S0002-9440(10)63693-0 PMC1868200

[bib4] Greijer AE, van der Wall E (2004) The role of hypoxia inducible factor 1 (HIF-1) in hypoxia induced apoptosis. J Clin Pathol 57: 1009–1014 15452150 10.1136/jcp.2003.015032 PMC1770458

[bib5] Hattori H, Tanaka M, Fukushima M, Sasaki T, Matsuda A (1996) Nucleosides and nucleotides. 158. 1-(3-C-ethynyl-beta-D-ribo-pentofuranosyl)-cytosine, 1-(3-C-ethynyl-beta-D-ribo-pentofuranosyl)uracil, and their nucleobase analogues as new potential multifunctional antitumor nucleosides with a broad spectrum of activity. J Med Chem 39: 5005–5011 8960561 10.1021/jm960537g

[bib6] Iizuka D, Inanami O, Matsuda A, Kashiwakura I, Asanuma T, Kuwabara M (2005) X irradiation induces the proapoptotic state independent of the loss of clonogenic ability in Chinese hamster V79 cells. Radiat Res 164: 36–44 15966763 10.1667/rr3393

[bib7] Inanami O, Iizuka D, Iwahara A, Yamamori T, Kon Y, Asanuma T, Matsuda A, Kashiwakura I, Kitazato K, Kuwabara M (2004) A novel anticancer ribonucleoside, 1-(3-C-ethynyl-beta-D-ribo-pentofuranosyl)cytosine, enhances radiation-induced cell death in tumor cells. Radiat Res 162: 635–645 15548113 10.1667/rr3268

[bib8] Kashiwakura I, Murakami M, Inanami O, Hayase Y, Takahashi TA, Kuwabara M, Takagi Y (2002) Effects of amifostine on the proliferation and differentiation of megakaryocytic progenitor cells. Eur J Pharmacol 437: 19–25 11864634 10.1016/s0014-2999(02)01270-0

[bib9] Kilic M, Kasperczyk H, Fulda S, Debatin KM (2007) Role of hypoxia inducible factor-1 alpha in modulation of apoptosis resistance. Oncogene 26: 2027–2038 17043658 10.1038/sj.onc.1210008

[bib10] Koizumi K, Shimamoto Y, Azuma A, Wataya Y, Matsuda A, Sasaki T, Fukushima M (2001) Cloning and expression of uridine/cytidine kinase cDNA from human fibrosarcoma cells. Int J Mol Med 8: 273–278 11494055

[bib11] Koukourakis MI, Giatromanolaki A, Sivridis E, Simopoulos C, Turley H, Talks K, Gatter KC, Harris AL (2002) Hypoxia-inducible factor (HIF1A and HIF2A), angiogenesis, and chemoradiotherapy outcome of squamous cell head-and-neck cancer. Int J Radiat Oncol Biol Phys 53: 1192–1202 12128120 10.1016/s0360-3016(02)02848-1

[bib12] Maehara Y, Nakamura H, Nakane Y, Kawai K, Okamoto M, Nagayama S, Shirasaka T, Fujii S (1982) Activities of various enzymes of pyrimidine nucleotide and DNA syntheses in normal and neoplastic human tissues. Gann 73: 289–298 6288502

[bib13] Matsuda A, Sasaki T (2004) Antitumor activity of sugar-modified cytosine nucleosides. Cancer Sci 95: 105–111 14965358 10.1111/j.1349-7006.2004.tb03189.x PMC11159627

[bib14] Murata D, Endo Y, Obata T, Sakamoto K, Syouji Y, Kadohira M, Matsuda A, Sasaki T (2004) A crucial role of uridine/cytidine kinase 2 in antitumor activity of 3′-ethynyl nucleosides. Drug Metab Dispos 32: 1178–1182 15280220 10.1124/dmd.104.000737

[bib15] Ogura A, Watanabe Y, Iizuka D, Yasui H, Amitani M, Kobayashi S, Kuwabara M, Inanami O (2008) Radiation-induced apoptosis of tumor cells is facilitated by inhibition of the interaction between Survivin and Smac/DIABLO. Cancer Lett 259: 71–81 17967504 10.1016/j.canlet.2007.09.017

[bib16] Peng XH, Karna P, Cao Z, Jiang BH, Zhou M, Yang L (2006) Cross-talk between epidermal growth factor receptor and hypoxia-inducible factor-1alpha signal pathways increases resistance to apoptosis by up-regulating survivin gene expression. J Biol Chem 281: 25903–25914 16847054 10.1074/jbc.M603414200 PMC3132567

[bib17] Semenza GL (2003) Targeting HIF-1 for cancer therapy. Nat Rev Cancer 3: 721–732 13130303 10.1038/nrc1187

[bib18] Shimamoto Y, Koizumi K, Okabe H, Kazuno H, Murakami Y, Nakagawa F, Matsuda A, Sasaki T, Fukushima M (2002) Sensitivity of human cancer cells to the new anticancer ribo-nucleoside TAS-106 is correlated with expression of uridine-cytidine kinase 2. Jpn J Cancer Res 93: 825–833 12149149 10.1111/j.1349-7006.2002.tb01325.x PMC5927072

[bib19] Vergis R, Corbishley CM, Norman AR, Bartlett J, Jhavar S, Borre M, Heeboll S, Horwich A, Huddart R, Khoo V, Eeles R, Cooper C, Sydes M, Dearnaley D, Parker C (2008) Intrinsic markers of tumour hypoxia and angiogenesis in localised prostate cancer and outcome of radical treatment: a retrospective analysis of two randomised radiotherapy trials and one surgical cohort study. Lancet Oncol 9: 342–351 18343725 10.1016/S1470-2045(08)70076-7

[bib20] Yasui H, Inanami O, Asanuma T, Iizuka D, Nakajima T, Kon Y, Matsuda A, Kuwabara M (2007) Treatment combining X-irradiation and a ribonucleoside anticancer drug, TAS106, effectively suppresses the growth of tumor cells transplanted in mice. Int J Radiat Oncol Biol Phys 68: 218–228 17448876 10.1016/j.ijrobp.2006.12.061

[bib21] Zhang Q, Zhang ZF, Rao JY, Sato JD, Brown J, Messadi DV, Le AD (2004a) Treatment with siRNA and antisense oligonucleotides targeted to HIF-1alpha induced apoptosis in human tongue squamous cell carcinomas. Int J Cancer 111: 849–857 15300796 10.1002/ijc.20334

[bib22] Zhang X, Kon T, Wang H, Li F, Huang Q, Rabbani ZN, Kirkpatrick JP, Vujaskovic Z, Dewhirst MW, Li CY (2004b) Enhancement of hypoxia-induced tumor cell death *in vitro* and radiation therapy *in vivo* by use of small interfering RNA targeted to hypoxia-inducible factor-1alpha. Cancer Res 64: 8139–8142 15548675 10.1158/0008-5472.CAN-03-2301

